# Two Distantly Spaced Basic Patches in the Flexible Domain of Huntingtin-Interacting Protein 1 (HIP1) Are Essential for the Binding of Clathrin Light Chain

**DOI:** 10.1155/2009/256124

**Published:** 2009-04-07

**Authors:** Joel A. Ybe, Mary E. Clegg, Melissa Illingworth, Claire Gonzalez, Qian Niu

**Affiliations:** Department of Biology, Indiana University, Simon Hall 405B, 212 S. Hawthorne Drive, Bloomington, IN 47405, USA

## Abstract

The interaction between HIP family proteins (HIP1 and HIP12/1R) and clathrin is fundamental to endocytosis. We used circular dichroism (CD) to study the stability of an HIP1 subfragment (aa468-530) that is splayed open. CD thermal melts show HIP1 468-530 is only stable at low temperatures, but this HIP1 fragment contains a structural unit that does not melt out even at 83°C. We then created HIP1 mutants to probe our hypothesis that a short hydrophobic path in the opened region is the binding site for clathrin light chain. We found that the binding of hub/LCb was sensitive to mutating two distantly separated basic residues (K474 and K494). The basic patches marked by K474 and K494 are conserved in HIP12/1R. The lack of conservation in *sla2p (S. cerevisiae)*, HIP1 from *D. melanogaster*, and HIP1 homolog ZK370.3 from *C. elegans* implies the binding of HIP1 and HIP1 homologs to clathrin light chain may be different in these organisms.

## 1. Introduction

Huntingtin-interacting protein 1 (HIP1) and its relative, HIP12/1R, contribute to the budding of clathrin-coated vesicles (CCVs) [1, 2]. The yeast homolog of HIP1, *sla2p*, is required for the development of CCVs in yeast [3, 4]. There are shared regions in HIP1 and HIP12/1R that mediate the binding of clathrin, but there are also sites that are unique to each HIP protein. For example, HIP1, but not HIP12/1R, has a clathrin box motif (_332_LMDMD ) which functions to bind the N-terminal beta propeller domain of clathrin [5]. HIP1 and HIP12/1R do not interact with adaptor protein 2 (AP2) in the same way. The AP2-binding FXDXF and DPF motifs (X denotes any amino acid) are only in HIP1 [5]. HIP12/1R apparently has a higher affinity for F-actin [6], suggesting that HIP1 and HIP12/1R play distinct roles in the formation of CCVs. A number of amino acids (L_486_ and R_487_ in the DLL_486_R_487_KN region) in the coiled-coil domain of HIP1 and HIP12/1R have been identified to impact the binding of clathrin light chain (CLC) [7], but we do not know if L_486_ and R_487_ constitute the entire CLC binding determinants in HIP proteins. We recently determined the crystal structures of two contiguous subfragments of the HIP1 coiled-coil domain (PDB files: 2NO2 and 2QA7) that span the clathrin light chain-binding region [8, 9]. The DLL_486_R_487_KN stretch was embedded in the splayed opened region that we first discovered in HIP1 482-586 (2NO2) [8] and later found in HIP1 371-481 (2QA7) [9]. In this research letter we define the inherent stability of a segment of HIP1 that contains the opened region and ask if electrostatic interactions help drive the binding of CLC. We generated an HIP1 subfragment (aa468-530) that spans the opened region for circular dichroism (CD) experiments to assess this domain's intrinsic stability. We then studied if two distantly spaced positively charged patches in the opened region played any role in the binding of CLC. Here we report that the HIP1 468-530 construct is unstable, but paradoxically a heat-resistant structural unit is present within this subfragment. We found that the two basic patches in the flexible part of HIP1 (centered on K474 and K494) are crucial for the binding of CLC. These electrostatic determinants are part of a solvent exposed hydrophobic surface that we previously argued was suitable for CLC.

## 2. Results and Discussion

### 2.1. Coiled-Coil Segment of HIP1 that Includes Determinants for Clathrin Light Chain Is Intrinsically Unstable

**Figure 1:**
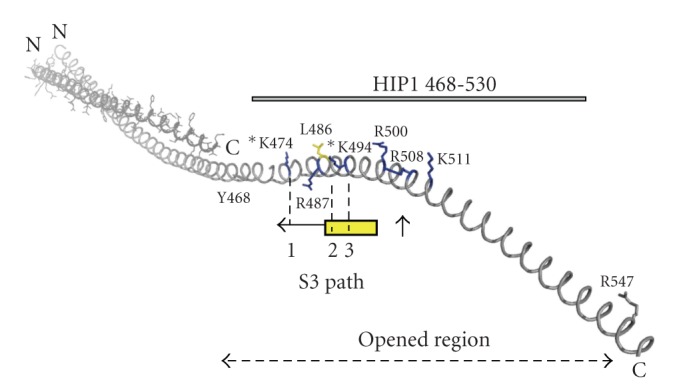
Structural features of the HIP1 clathrin light chain-binding site. Two distantly separated basic amino acids in the opened region of HIP1 mediate the binding of clathrin light chain. The assembled model was generated using PDB files 2NO2 [8] and 2QA7 [9]. For the sake of clarity, we only show a portion of the second HIP1 helix (dark grey) that stops before Y468. The yellow bar marks the position of the solvent exposed hydrophobic S3 path [8]. The new data in Figure 3 indicate that K474 is a strong determinant for binding and imply that S3 path begins before the DLL_486_R_487_KN region. The N- and C-termini of the HIP1 crystal structure are labeled N and C. The numbers 1–3 along the yellow bar mark the position of amino acids that control the binding of clathrin light chain (position 1: K474; position 2: L486 and R487 reported by the McPherson group [7]; and position 3: K494). K474 and K494 are ~50 Å apart. R500, R508 or K511 (see arrow) do not participate in binding and therefore define the boundary of the light chain-binding site. The HIP1 468-530 subfragment used in the CD studies in Figure 2 spans across an opened region of the HIP1 coiled coil in our 2NO2 and 2QA7 crystal structures. The HIP1 model was created using PyMol (http://www.pymol.org).

**Figure 2:**
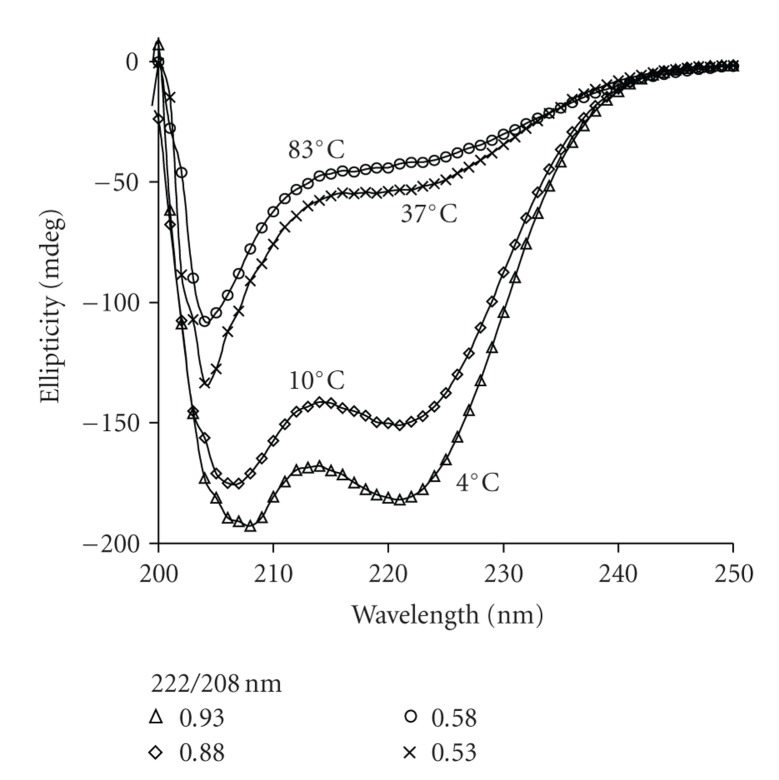
HIP1 468-530 subfragment in the opened region is stable only at low temperature. Equilibrium far UV CD scans were performed at 4°C (open triangle), 10°C (open diamond), 37°C (cross), and 83°C (open circle). The position of the 222 nm signal did not change with temperature, but the 208 nm signal shifted as the sample was heated. The 222/208 nm ratios were calculated from the raw CD data. Because the signal below 200 nm was noisy, we did not attempt to get the helix content from the CD data.

 The binding of CLC to HIP1 requires L486 or R487 [7] (human HIP1 numbering (accession number NP 005329), conserved in HIP12/1R). We recently located L486 and R487 to a flexible segment of HIP1 (position 2, Figure 1), in S3 path [8]. Here we used CD to probe the stability of HIP1 468-530 (marked by the grey strip in Figure 1, between Y468 and R547), a coiled-coil segment that has the S3 path. At 4°C the CD profile was helical, indicated by the double minimum at 208 and 222 nm (Figure 2). The ratio of intensities at 222 and 208 nm can be used to distinguish coiled coils from isolated helices (≥1 for coiled coils and <0.86 for isolated helices [10, 11]). The ratio values in Figure 2 suggest that HIP1 468-530 shifts from a mixture of isolated helices and coiled coils at low temperatures to isolated helices at 37°C. We point out that the 37°C profile is almost identical to the 83°C scan, suggesting that HIP1 468-530 contains a heat-stable element. We are presently doing NMR experiments to map out the extent of the heat unstable portion of HIP1 468-530.

### 2.2. Basic Patches Centered on K474 and K494 in HIP1 Are Essential for Binding Clathrin Light Chain

**Figure 3:**
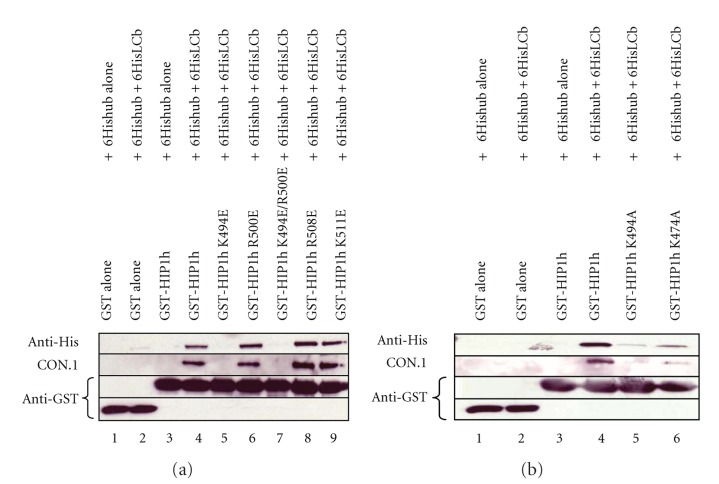
Two basic patches in HIP1 are critical for binding clathrin light chain. Western blots were performed to analyze GST-pulldown experiments. Samples were run on the same gel, but after transfer, the membrane was cut apart and blotted with the appropriate antibody. Bovine clathrin heavy chain (aa1074-1675, Hub) with an N-terminal histidine-tag was blotted with a commercial anti-His monoclonal antibody (Anti-His). The neuronal form of clathrin light chain b (LCb) had an N-terminal histidine-tag, but we used CON.1 to identify LCb. The GST alone and GST-HIP1h constructs were detected using a commercial monoclonal antibody against GST (Anti-GST). Panel A: lanes 1 and 2: GST alone negative controls show that there are no nonspecific interactions with 6Hishub alone or 6Hishub + 6HisLCb. Lane 3: wild type GST-HIP1h pulldown of 6Hishub alone (no bound LCb) indicates that the interaction needs light chain. Lane 4: positive control, wild type GST-HIP1h with 6Hishub/6HisLCb. Lanes 5–9: pulldowns of HIP1h single and double mutants with 6Hishub/6HisLCb. Panel B: lanes 1–3 are controls as described in panel A. Lane 4: positive control, wild type GST-HIP1h with 6Hishub/6HisLCb. Lane 5: GST-HIP1h K494A with 6Hishub/6HisLCb. Lane 6: GST-HIP1h K474A with 6Hishub/6HisLCb.

 We investigated if a series of basic patches in the opened region could participate in binding CLC. K494 (see position 3, Figure 1) is in S3 path (yellow bar, Figure 1) previously described [8] and is followed by a cluster of basic residues (indicated by the arrow in Figure 1). K474 (position 1) is located before the DLL_486_R_487_KN region, close to the part of HIP1 that mediates the binding of Huntingtin interacting protein 1 interactor (HIPPI) [9]. To probe if those in S3 path contributed, we made 5 GST-HIP1h (HIP1h is aa370-644) 370-644 mutants (K494A, K494E, R500E, R508E, and K511E). We performed GST pulldowns to evaluate the binding of 6Hishub/6HisLCb (hub is central third of clathrin (aa1074-1675) [12] and 6HisLCb is N-terminally histidine-tagged bovine clathrin light chain b with the neuronal insert). It is important to study 6HisLCb that is bound to 6Hishub to closely mimic how HIP1 interacts with clathrin baskets in cells. Every GST pulldown was done at least three times, using freshly isolated proteins and charged GST beads each time. Clathrin hub (N-terminally histidine tagged) was detected by western blotting with a commercial histidine tag antibody. LCb was blotted with CON.1 monoclonal antibody and GST-HIP1h constructs were visualized with a commercial GST antibody. The anti-GST bands in Figures 3(a) and 3(b) showed that the GST-HIP1h levels were balanced (loading control). The negative controls in lanes 1–3 in Figure 3(a) and lanes 1–3 in Figure 3(b) show that the GST signals from each binding experiment were not random interactions, but reflected true binding events. The level of 6Hishub/6HisLCb captured by GST-HIP1h is shown in lane 4 in Figures 3(a) and 3(b), and as expected, required bound LCb (compare lanes 3 and 4 in Figures 3(a) and 3(b)). We did not remove the histidine tag on LCb used for purification because control experiments showed that the tag did not interfere with the GST pulldowns (data not shown). The binding of 6Hishub/6HisLCb was significantly blocked when HIP1h K494 was replaced with glutamic acid (K494E mutant, lane 5 in Figure 3(a)) or with alanine (lane 5, Figure 3(b)). In contrast, we saw no detectable impact when R500, directly above K494 (see Figure 1 for location), was changed to glutamic acid (see lane 6, Figure 3(a)) or alanine (data not shown). Consistent with data in lanes 5 and 6 in Figure 3(a), the K494E/R500E double mutant did not bind 6Hishub/6HisLCb (lane 7, Figure 3(a)). Next we evaluated a group of basic residues close to the C-terminal end of S3 path (indicated by an arrow in Figure 1). Lanes 8 and 9 in Figure 3(a) show that the R508E and K511E charge flip mutants bound 6Hishub/6HisLCb similar to the wild type control (compare lane 4 with lanes 8-9 in Figure 3(a)). We conclude from these data that K494 located in the hydrophobic S3 path is required to bind CLC, but R500, R508, and K511 around this path do not participate. This suggests that the CLC binding site does not go beyond the boundaries of S3 path that is defined by R500, R508, and K511.

The assembled HIP1 model (2NO2 joined to 2QA7) in Figure 1 shows how the positive patch centered on K474 is oriented relative to K494 (separated by ~50 Å). Our data in Figure 3(b) show a dramatic drop in the binding of 6Hishub/6HisLCb when K474 was mutated to alanine (see lanes 4 and 6 (K494A in lane 5 for comparison). This result demonstrates for the first time that K474 upstream the DLL_486_R_487_KN region is necessary for binding 6Hishub/6HisLCb and suggests that the CLC binding site may be more extensive than previously thought. We predict that the two basic patches in HIP1 we have defined here are also present in HIP12/R because K474 and K494 (HIP1 numbering) are both conserved in this protein. Finally, we looked if K474, R487, and K494 were conserved in HIP1 from veterbrates *Mus musculus*, *Rattus norvegicus*, *Xenopus laevis*, *Drosophila melanogaster*, and in *sla2p* (HIP1 homolog from *Saccharomyces cerevisiae*) and ZK370.3 (HIP1 homolog from *Caenorhabditis elegans*). This analysis showed that K474 was conserved in all the vertebrates but was V in *C. elegans*, L in *D. melanogaster*, and M in sla2p. R487 was conserved in all the vertebrates but was E in both *D. melanogaster* and *sla2p* and T in *C. elegans*. K494 was conserved in all vertebrates and invertebrate HIP1 proteins, except *sla2p* (K474 (Q in *S. cerevisiae*), R487 (T), and K494 (D)). The natural mutations in *sla2p*, HIP1 from *D. melanogaster*, and ZK370.3 from *C. elegans* could mean that the binding of HIP1 and HIP1 homologs to clathrin light chain in these organisms is different.

## 3. Materials and Methods

### 3.1. Materials

Triton X-100, Tween-20, beta-mercaptoethanol (*β*ME), TRIZMA base, and BIS-TRIS were from Sigma-Aldrich (St. Louis, Mo, USA). Sodium phosphate dibasic (Na_2_HPO_4_) was from EMD Chemicals (Gibbstown, NJ, USA); Luria broth was from EMD Biosciences (Sparks, Md, USA); tris(2-carboxyethyl)-phosphine was from Sigma-Aldrich. Pfu turbo was from Stratagene (La Jolla, Calif, USA) and primers were from Integrated DNA Technologies (Coralville, Iowa, USA). The Pierce Coomassie Plus Bradford reagent kit was purchased from Fisher Scientific (Hanover Park, Ill, USA). Chromatography resins, columns, and standards were purchased from GE Healthcare (Piscataway, NJ, USA). CON.1 antibody was bought from Covance (Cumberland, Va, USA); restriction grade thrombin and the anti-His antibody were obtained from Novagen (La Jolla, Calif, USA). Coomassie G-250 stain and Immun-Star chemiluminescent kit were from Bio-Rad Laboratories (Hercules, Calif, USA).

### 3.2. Construction of GST-HIP1h Mutants and 6HisLCb

The plasmid encoding the original N-terminal GST tagged HIP1h 370-644 was a gift from the McPherson group. The various GST-HIP1h mutants used in this work were created using the QuikChange mutagenesis protocol (Stratagene). The sequence was confirmed by DNA sequencing (IMBI, Indiana University) and then transformed into Rosetta 2 (DE3) pLysS cells (Novagen). Standard cloning was used to insert neuronal LCb DNA in *p*ET15b to generate 6HisLCb. The recombinant 6HisLCb plasmid was transformed into BL21 (DE3) pLysS cells for overexpression.

### 3.3. Protein Overexpression and Purification

The recombinant GST-HIP1h constructs were grown at 37°C in 1 L Luria broth (LB) to an O.D. 600 of 0.5–0.8 units. The incubation temperature was dropped to 30°C cells and protein expression was induced with IPTG (100 *μ*g/mL final concentration). Cells were harvested after 3 hours at 30°C and bacterial pellets were frozen at −80°C before use. Bacterial pellets were resuspended in 50 mL of 1X PBS (10 mM Na_2_HPO_4_, 1.8 mM KH_2_PO_4_ (pH 7.3), 140 mM NaCl, 2.7 mM KCl, supplemented with 0.25 mL of 1 M DTT, 0.25 mL of protease inhibitor cocktail (Sigma), and 2 mL of PMSF (17.4 mg/mL in 2-propanol)). After sonication, 2.5 mL of 20% (v/v) Triton X-100 was added and the lysate was rotated at room temperature for ~30 minutes. The crude bacterial lysate was spun at 12 000 g (4°C) for 10 minutes. The supernatant was mixed with ~5 mL of glutathione Sepharose 4B (Amersham) resin suspended in PBS. The GST-HIP1h constructs were eluted from the column with 50 mL of 3 mg/mL L-glutathione (sigma) in (PBS) at pH 8.0 and dialyzed overnight against the same buffer. For CD experiments the purification protocol was modified so that we can cleave the GST tag in the GST column. After bacterial lysate was added to GST beads, the mixture was rocked at room temperature for 1-2 hours. The beads were spun down at 500 g at 4°C for 5 minutes and the supernatant was poured off. The wet beads were transferred to a column and washed slowly with 110 mL 1X PBS and then 1 unit thrombin per mg of protein was diluted into 3.5 mL of 1X PBS and added to the column. After digestion, the HIP1h constructs were further purified on Superdex 75 column (GE Healthcare) equilibrated with 1X PBS (at room temperature). Column fractions were pooled and dialyzed at 4°C against 10 mM potassium phosphate buffer at pH 7.9.

The clathrin hub construct was purified as previously described [13] and the 6HisLCb was purified in a single step using the nickel affinity resin. After the crude lysate was added to charged Sepharose nickel resin and incubated at 4°C for 10 minutes, the beads were gently spun down and washed with 25 mL of 10 mM Na_2_HPO_4_, 10 mM imidazole, 0.5 M NaCl, pH 7.4 buffer (buffer A). The beads were then washed with 30 mL of buffer A that contained 0.5 M imidazole. The 6HisLCb was eluted off the column with buffer A that contained 205 mM imidazole. EDTA was added to the sample (1 mL 0.5 M EDTA per 10 mL of protein) and dialyzed against 10 mM tris, pH 7.9 overnight at 4°C (dialysis buffer includes BME, and a cocktail of protease inhibitor).

### 3.4. CD Measurements

Purified HIP1 468-530 was diluted with 10 mM potassium phosphate buffer at pH 7.9 to 0.5 mg/mL for CD measurements at different temperatures. CD data was collected using a Jasco J-175 circular dichroism spectropolarimeter with thermally controlled sample cells. The first CD scan was taken at 4°C and then the temperature was changed to the indicated temperatures in Figure 2. The sample was allowed to sit at each indicated temperature for several minutes before taking the CD scan.

### 3.5. GST Pull Down Assays

Glutathione Sepharose 4B resin (GE Healthcare 17-0756-01) was washed three times with 1 mL of PBS. Protein concentrations were determined by Bradford assays (Pierce 23236). Equal molar amounts of GST and GST-Hip1h proteins (1  *μ*M) and 1 mL of PBS were added to resin and incubated on a rotating platform at 4°C for 1 hour. Unbound proteins were removed by washing three times with binding buffer (50 mM Tris, 200 mM KCl, 1 mM EDTA, 1% Triton X-100, 50 mM imidazole, 0.5 mg/mL ovalbumin, pH 8.0). 6HisLCb and Hub alone were combined in a 3:1 ratio and allowed to incubate at 4°C for at least 30 minutes. 25 *μ*L of GST or GST-Hip1h bound beads, 0.4 nmol of Hub Alone or 1.9 nmol of Hub 6HisLCb complex, 375 *μ*L of room temperature binding buffer were added to illustra MicroSpin columns (GE Healthcare 27-3565-01) and incubated on rotator at 4°C for 1 hour. Beads were washed six times with 0.5 mL of binding buffer + 16 mM imidazole and supernatant removed by centrifugation. After final wash, 55 *μ*L of 2x SDS gel loading buffer was added to beads. Spin columns were closed placed in 1.7 mL Eppendorf tubes, heated for 10 minutes at 80–90°C, and spun down to collect samples.

### 3.6. Western Blots

The bound proteins were resolved by SDS-PAGE and analyzed by standard western blotting. After transfer the nitrocellulose membrane was stained with Ponceau stain and cut to separate proteins for blotting. Hub was detected with anti-His monoclonal antibody (Novagen 70796-3), GST and GST-Hip1h were detected with anti-GST monoclonal antibody (Covance MMS-112P), and 6HisLCb was detected with clathrin light chain monoclonal antibody (CON.1) (Covance MMS-423P). Binding was detected with Immun-Star chemiluminescent protein detection system (BioRad 170-5010).

## References

[1] Engqvist-Goldstein ÅEY, Kessels MM, Chopra VS, Hayden MR, Drubin DG (1999). An actin-binding protein of the Sla2/Huntingtin interacting protein 1 family is a novel component of clathrin-coated pits and vesicles. *The Journal of Cell Biology*.

[2] Mishra SK, Agostinelli NR, Brett TJ, Mizukami I, Ross TS, Traub LM (2001). Clathrin- and AP-2-binding sites in HIP1 uncover a general assembly role for endocytic accessory proteins. *The Journal of Biological Chemistry*.

[3] Sun Y, Kaksonen M, Madden DT, Schekman R, Drubin DG (2005). Interaction of Sla2p’s ANTH domain with PtdIns(4,5)P2 is important for actin-dependent endocytic internalization. *Molecular Biology of the Cell*.

[4] Newpher TM, Idrissi F-Z, Geli MI, Lemmon SK (2006). Novel function of clathrin light chain in promoting endocytic vesicle formation. *Molecular Biology of the Cell*.

[5] Brett TJ, Traub LM, Fremont DH (2002). Accessory protein recruitment motifs in clathrin-mediated endocytosis. *Structure*.

[6] Brett TJ, Legendre-Guillemin V, McPherson PS, Fremont DH (2006). Structural definition of the F-actin-binding THATCH domain from HIP1R. *Nature Structural and Molecular Biology*.

[7] Legendre-Guillemin V, Metzler M, Lemaire J-F (2005). Huntingtin interacting protein 1 (HIP1) regulates clathrin assembly through direct binding to the regulatory region of the clathrin light chain. *The Journal of Biological Chemistry*.

[8] Ybe JA, Mishra S, Helms S, Nix J (2007). Crystal structure at 2.8Å of the DLLRKN-containing coiled-coil domain of Huntingtin-interacting protein 1 (HIP1) reveals a surface suitable for clathrin light chain binding. *Journal of Molecular Biology*.

[9] Niu Q, Ybe JA (2008). Crystal structure at 2.8Å of Huntingtin-interacting protein 1 (HIP1) coiled-coil domain reveals a charged surface suitable for HIP1 protein interactor (HIPPI). *Journal of Molecular Biology*.

[10] Lau SYM, Taneja AK, Hodges RS (1984). Synthesis of a model protein of defined secondary and quaternary structure. Effect of chain length on the stabilization and formation of two-stranded *α*-helical coiled-coils. *The Journal of Biological Chemistry*.

[11] McNamara C, Zinkernagel AS, Macheboeuf P, Cunningham MW, Nizet V, Ghosh P (2008). Coiled-coil irregularities and instabilities in group A Streptococcus M1 are required for virulence. *Science*.

[12] Liu S-H, Wong ML, Craik CS, Brodsky FM (1995). Regulation of clathrin assembly and trimerization defined using recombinant triskelion hubs. *Cell*.

[13] Ybe JA, Perez-Miller S, Niu Q, Coates DA, Drazer MW, Clegg ME (2007). Light chain C-terminal region reinforces the stability of clathrin heavy chain trimers. *Traffic*.

